# Involvement of mammalian SoLute Carriers (SLC) in the traffic of polyamines

**DOI:** 10.3389/fmolb.2024.1452184

**Published:** 2024-07-25

**Authors:** Lorena Pochini

**Affiliations:** ^1^ Laboratory of Biochemistry, Molecular Biotechnology and Molecular Biology, Department DiBEST (Biologia, Ecologia, Scienze Della Terra), University of Calabria, Rende, Italy; ^2^ Institute of Biomembranes, Bioenergetics and Molecular Biotechnologies (IBIOM), National Research Council (CNR), Bari, Italy

**Keywords:** SLC18B1, SLC22A4, OCTN1, cancer, neuronal disorders, spermine, spermidine, putrescine

## Abstract

Polyamines interact with different molecular targets to regulate a vast range of cellular processes. A network of enzymes and transport systems is crucial for the maintenance of polyamine homeostasis. Indeed, polyamines after synthesis must be distributed to the various tissues and some intracellular organelles. Differently from the well characterized enzymes devoted to polyamine synthesis, the transport systems are not unequivocally identified or characterized. Besides some ATPases which have been identified as polyamine transporters, much less is known about solute carriers (SLC) involved in the transport of these compounds. Only two SLCs have been unequivocally identified as polyamine transporters: SLC18B1 (VPAT) and SLC22A4 (OCTN1). Transport studies have been performed with cells transfected with the cDNAs encoding the two and other SLCs or, in the case of OCTN1, also by *in vitro* assay using proteoliposomes harboring the recombinant human protein. According to the role proposed for OCTN1, polyamines have been associated with prolonged and quality of life. This review provides an update on the most recent findings concerning the polyamine transporters or the prediction of the putative ones.

## 1 Introduction

Spermine, spermidine and putrescine are the most abundant polyamines in mammals, representing ubiquitous and essential molecules for human cell metabolism [for a complete list of important polyamines see (Rieck et al., 2022)]. Polyamines are organic polycations with at least two charged groups responsible for non-specific interactions with negatively charged macromolecules. Site-specific, orthosteric, allosteric interactions and covalent binding with different molecular targets have been reported as well (Zimmermann et al., 2023) explaining the different functions performed by polyamines which are involved in the regulation of a vast range of cellular processes (Luo et al., 2023; Vrijsen et al., 2023) as well as in health-protection effects (Madeo et al., 2018; Nilsson and Persson, 2019). Very importantly, the homeostasis of polyamines requires a network of enzymes and transport systems (Vrijsen et al., 2023). Indeed, polyamines do not only originate from cell biosynthesis but also from the diet and the gut microbiota. Therefore, from the gut, polyamines are distributed to the tissues via the bloodstream and then taken up in cells by specific transporters (Vrijsen et al., 2023). The concerted action of the enzymes and transporters which is dictated by different kinetic properties together with different tissue localizations, allows polyamines to reach different levels in different tissues. Dysregulation of polyamine homeostasis strongly associates with human diseases (Azfar et al., 2022). Most of the literature addresses the interest towards these molecules in cancer and nervous system physiology/pathology. Several oncogenic pathways led to the dysregulation of polyamine metabolism: elevated polyamine levels are necessary for transformation and tumor progression (Casero et al., 2018). Ornithine decarboxylase 1 (ODC), the rate-limiting enzyme of polyamine synthesis, is transcriptionally activated by the oncogene MYC. Inhibiting ODC activity largely reduces polyamine synthesis and the incidence of colorectal cancer supporting the role played by polyamine in cancer (Du and Han, 2021). Very recently a polyamines’ ferroptosis-sensitizing effect has been described (Bi et al., 2024). Polyamine transport activity exists in several human colorectal cancer cell lines; however, large gaps in the knowledge concerning the precise molecular mechanisms of mammalian polyamine transport exist (Corral and Wallace, 2020). Indeed, polyamine transporters are still referred to as, the so called, “Polyamine Transport System (PTS)” even though different molecular entities are involved in transport. Polyamine transport mediated by membrane transporters is attributed mainly to the activity of P-type ATPases (Azfar et al., 2022) and only in part to SLCs. ATP13As have recently been addressed (Croucher and Fleming, 2023; Houdou et al., 2023; Mu et al., 2023; van Veen et al., 2023; Liu et al., 2024) and new strategies for studying polyamine transport have been set up, such as the employment of fluorescent polyamine probes (Vanhoutte et al., 2018). SLC22A4 (OCTN1) and SLC18B1 (VPAT) are the best acknowledged transporters for polyamines. Filling this gap is very important since the SLC superfamily is currently positioned at the centre of novel pharmacological targeting strategies and drug development. This review provides an update on the SLC-mediated transport of polyamines (Table 1).

**TABLE 1:** Putative substrates of polyamine SLC transporters and transport mechanisms.

Gene/Protein	Putative PA (Substrate)	Localization of PA transport in normal/cancer tissues	Mechanism	References
SLC6A2/NET	Spd	ND (Computational analysis)	Sodium dependent cotransport	Shen et al. (2023)
SLC6A4/5-HTT	Spd	pancreatic acinar cells	Sodium dependent cotransport	Shen et al. (2023)
SLC6A14/ATB^0,+^	Arg	Low level in normal tissues/Breast, Colorectal, Cervical cancer	Sodium and Chloride dependent cotransport	Karunakaran et al. (2011), Du and Han (2021)
SLC7A1/CAT1	Arg, Put, Spd	Aorta, NT, RPE, BM/Colorectal cancer	Uniport	Kaneko et al. (2007), Grossi et al. (2014), Forte et al. (2018), Du and Han (2021), Rieck et al. (2022), Gonzalez-Menendez et al. (2023)
SLC7A2/CAT2	Arg, Agm	Macrophages, NT	Uniport	Chaturvedi et al. (2010), Molderings and Haenisch (2012), Bernstein et al. (2020)
SLC7A6/y^+^LAT2	Put, Arg	Fibroblasts, Retinal pigment epithelial cells/Caco‐2 cells, N	Uniport	Saulnier Sholler et al. (2015), Rotoli et al. (2020)
SLC7A7/y^+^LAT1	Put, Arg	Monocytes-macrophages/Caco‐2 cells, N	Uniport	Saulnier Sholler et al. (2015), Rotoli et al. (2020)
SLC12A8A/CCC9a	Arg, Spm	B and Lung cancer	Chloride dependent cotransport	Daigle et al. (2009), Moriyama et al. (2020), Zhang et al. (2021), Zheng et al. (2023)
SLC18B1/VPAT	Spm, Spd	NT, A, P, K, T, L, B, mast cells, macrophages, MEG-01 and platelets	Proton dependent antiport	Hiasa et al. (2014), Takeuchi et al. (2017), Fredriksson et al. (2019), Park et al. (2019), Moriyama et al. (2020), Uehara et al. (2024)
SLC21/OATP4C1	Arg	K	Uniport	Taghikhani et al. (2019)
SLC22A1	Spm,Agm, Spd	K, NT, L	Uniport	Grundemann et al. (2003), Nies et al. (2011), Winter et al. (2011), Sala-Rabanal et al. (2013), Schophuizen et al. (2013), Akanuma et al. (2017), Morris et al. (2017)
SLC22A2	Spm, Agm, Put,Spd	K, NT, L	Uniport	Grundemann et al. (2003), Nies et al. (2011), Winter et al. (2011), Sala-Rabanal et al. (2013), Schophuizen et al. (2013), Higashi et al. (2014), Akanuma et al. (2017), Morris et al. (2017), Cunha et al. (2022)
SLC22A3	Spm, Agm, Spd	K, NT	Uniport	Grundemann et al. (2003), Nies et al. (2011), Sala-Rabanal et al. (2013), Schophuizen et al. (2013), Higashi et al. (2014), Akanuma et al. (2017), Morris et al. (2017)
SLC22A4	Spd,Spm	K, I, NT, IC, A/Colorectal cancer	Uniport	Pochini et al. (2012), Masuo et al. (2018), Ben Mariem et al. (2024), Pochini et al. (2024)
SLC22A6/OAT1	Spd/Spm	K	Uniport	Ahn et al. (2011), Liu et al. (2016)
SLC22A16/CT2	Spd	T, BM/Testicular cancer, Lymphoma	Uniport	Aouida et al. (2010)
SLC22A18	Spd	Melanocytes	—	Brito et al. (2022)
SLC43A1	Put, Spd	VSMC	Uniport	Grossi et al. (2014)
SLC44A1/rCTL1	Put	BRB	Uniport	Tega et al. (2023)
SLC47A1/MATE1	Agm	K,L	Proton dependent antiport	Winter et al. (2011), Higashi et al. (2014)

Agm, Agmatine; Arg, Arginine; Spd, Spermidine; Spm, Spermine; PA, Polyamine; Put, Putrescine; A, Airway; B, Bladder; BM, Bone Marrow; BRB, Blood/Retinal Barrier; K, Kidney; I, intestine; IC, Immune cells; L, Liver; N, Neuroblastoma; ND, not determined; NT, nervous tissue; P, Placenta; RPE, Retinal pigment epithelial cells; T, Testis; VSMC, vascular smooth muscle cells.

## 2 SoLute carriers (SLCs) and polyamine transport

The SLC superfamily is the largest group of membrane transporter proteins. It includes 65 subfamilies with more than 500 members (Pizzagalli et al., 2021). They are responsible for the transport of a wide array of endogenous molecules and drugs and play a crucial role in human pathologies. Accordingly, the list of SLC transporters expressed in different tumors includes polyamine transporters (Bharadwaj et al., 2024). The SLC polyamine transporters known to date are localized to the plasma, lysosomal and mitochondrial membranes (Toninello et al., 1992; Moriyama et al., 2020). Indeed, in mitochondria, polyamines may play a role in regulating energy metabolism and mitochondrial gene expression; thus, they must enter within these organelles (Toninello et al., 1992; Grancara et al., 2014). The SLC superfamily includes the majority of polyamine transporters. The transport process is strongly regulated: when polyamine concentration decreases, transport increases. Moreover, the transporter activity is regulated by a feedback mechanism based on the immediate synthesis of antizyme which is a protein that blocks polyamine uptake in the presence of increased intracellular levels of polyamines (Lian et al., 2022; Lodeserto et al., 2022). The SLCs involved in polyamine transport are so far considered putative except for SLC18B1 and OCTN1 which are the sole validated polyamine transporters (Pochini et al., 2012; Hiasa et al., 2014; Masuo et al., 2018; Moriyama et al., 2020). The state of the art on individual polyamine SLCs is reported. SLCs which mediate the transport of arginine, thus being indirectly involved in polyamine homeostasis are also mentioned.

### 2.1 SLC3

SLC3A2 has been identified as the key transporter involved in polyamine uptake in neuroblastoma (Gamble et al., 2019). However, SLC3A2 has not an intrinsic transport activity but it mediates the trafficking of other transport proteins to the cellular membrane (Fotiadis et al., 2013; Scalise et al., 2021). SLC3A2 is a glycoprotein which forms heterodimers with various members of the SLC7 family. Therefore, it is important to identify the component of the heterodimers with SLC3A2 responsible for polyamine transport activity. Both the components of the heterodimer can be effective therapeutic targets (Khan et al., 2021; Eom et al., 2022).

### 2.2 SLC6

Computational analysis revealed that spermidine has a high affinity for SLC6A4 (5-HTT, SERT), the sodium-dependent serotonin transporter and SLC6A2 (NET), the sodium-dependent noradrenaline transporter (Shen et al., 2023). Cellular thermal shift assay revealed spermidine binding to SLC6A4 in pancreatic acinar cells. Moreover, a further member of this family, SLC6A14 (ATB^0,+^), was revealed to be one of the main transporters responsible for arginine transport, and was found overexpressed in colorectal cancer (Karunakaran et al., 2011; Du and Han, 2021).

### 2.3 SLC7

SLC7A1 and A2 (CAT1 and 2, system y^+^) are sodium independent arginine transporters (Jungnickel et al., 2018; Hobbach and Closs, 2020; Du and Han, 2021). CAT2 exists in two isoforms, CAT-2A and -2B, which differ in their affinity for arginine. L-arginine uptake in macrophages has been attributed to CAT2B (Chaturvedi et al., 2010). Both CAT1 and CAT2 are widely expressed in rat and human brains. Thus, they would play a role in neurodegenerative disorders (Bernstein et al., 2020). The blood–brain barrier (BBB) has been reported to be impermeable to polyamines (Weiss et al., 2023). Consequently, the brain depends on the activity of CAT1 for the transport of arginine which is used for polyamine biosynthesis in the brain (Rieck et al., 2022). Arginine uptake and its catabolism to spermidine would be involved in controlling erythroid differentiation (Gonzalez-Menendez et al., 2023). CAT1 would be involved in aortopathy progression (Forte et al., 2018). Putrescine, the monoacetylspermidines and diacetylspermine are all substrates for SLC3A2/Y^+^LAT (Saulnier Sholler et al., 2015). Monocytes/macrophages express high levels of SLC7A7 (y^+^LAT1) which would be the main responsible for arginine transport and SLC7A6 (y^+^LAT2) in fibroblasts (Rotoli et al., 2020). In polarized epithelia, such as renal and Caco‐2 cells (basolateral side) the system y^+^L would mediate arginine efflux in exchange with leucine and sodium (Rotoli et al., 2020). In melanocytes, SLC7A1 has been found downregulated by spermidine treatment. It would represent a strategy to maintain ideal cytosolic levels and avoid cytotoxic effects (Brito et al., 2022). In this context, spermidine has been reported as a promising compound for the treatment of hypopigmentation disorders supporting the stability of melanogenesis-related proteins.

### 2.4 SLC12

SLC12A8 belongs to a large family including 9 isoforms (Moreno et al., 2023). It is linked to certain cancers (Zhang et al., 2021), but the precise role of SLC12A8 in this context is unknown (Zheng et al., 2023). The splice variant SLC12A8A (CCC9a) was shown to be responsible for polyamine transport (Daigle et al., 2009; Zahedi et al., 2019). Very recently, the role of SLC12A8 as a polyamine transporter has been questioned (Sekhar et al., 2022).

### 2.5 SLC18

SLC18B1 (VPAT) is a polyspecific widely distributed transporter, performing a H^+^/polyamine antiport (Hiasa et al., 2014; Moriyama et al., 2020). It is mainly localized to endomembrane organelles relying on an H^+^ gradient created by V-ATPases. His-tagged VPAT was expressed in insect cells, purified, and co-reconstituted. Two different substrate binding sites were identified for spermine and spermidine, respectively (Hiasa et al., 2014). Interestingly, acetylcholine is also a substrate (Moriyama et al., 2020), transported with low affinity as previously found for another SLC polyamine transporter (Pochini et al., 2012). The agmatine transport remains to be characterized (Moriyama et al., 2020). SLC18B1 localizes with synaptic vesicles in neurons and synaptic-like microvesicles in astrocytes (Hiasa et al., 2014). It has been found responsible for the vesicular storage of spermine and spermidine in novel secretory granules that differ from histamine- and serotonin-containing granules and is involved in the vesicular release of these polyamines from mast cells (Takeuchi et al., 2017). Accordingly, the human megakaryoblastic cell line MEG-01 and platelets express VPAT (Uehara et al., 2024). The polyamine transport has also been explored *in vivo* employing the knock-out mouse model which was characterized by reduced polyamine content in neurons. Defects in learning and memory were observed (Fredriksson et al., 2019). In the field of diagnostics, SLC18B1 has been shown to be responsible for the transport of a novel optical imaging probe, CDg16 (Fredriksson et al., 2019), which could be used as a diagnostic tool for inflammation (Park et al., 2019).

### 2.6 SLC22

This family includes several members which are or may be polyamine transporters mediating the transport of anions, cations or zwitterions across the plasma or some intracellular membranes (Pizzagalli et al., 2021; Jamshidi and Nigam, 2022).

#### 2.6.1 SLC22A1-3

SLC22A1-3 (OCT1, 2 and 3) are organic cation transporters; they play roles in several human pathologies (Nies et al., 2011). Both human and rodent OCT2 and OCT3 are expressed in neurons and glial cells. On choroid plexus epithelial cells, a minor contribution of OCT3 to spermine elimination from the cerebrospinal fluid has been described. At the blood–brain barrier (BBB) and blood–cerebrospinal fluid (CSF) barrier (BCSFB) transporters different from OCTs, have been suggested (Akanuma et al., 2017). Transport of agmatine by the rat and human OCTs isoforms OCT2 and OCT3 has been demonstrated in HEK293 (Grundemann et al., 2003); whereas in human glioma SK-MG-1 cells, it has been suggested that it is very unlikely that OCTs and OCTNs would be involved in agmatine transport (Molderings and Haenisch, 2012). HEK293 transfected with hOCT1 or hOCT2 cDNA have been employed to assay [^3^H]agmatine and [^3^H]putrescine transport (Winter et al., 2011); hOCT2 was identified as a transporter for agmatine. OCTs spermidine transport activity has been measured in *Xenopus* oocytes expressing mammalian (mouse, rat) OCTs (Sala-Rabanal et al., 2013). In HEK293 cells, putrescine, agmatine and spermidine uptake by hOCTs, hOCTNs and hMATEs (see Sections 2.6.2 and 2.8) was also investigated (Higashi et al., 2014): significant putrescine, agmatine and spermidine uptake by hOCT2 was observed; Through trans-stimulation assay, agmatine has been identified as a global trans-stimulator of OCTs (Lefevre et al., 2021). Residues within the OCTs putative hydrophobic cleft that are not conserved in OCT3 were mutated to their corresponding OCT1 counterparts. Polyamines interacted poorly with the wild-type OCT3s but strongly with the mutants, as they do with OCT1, thus indicating that OCT1 might be involved in polyamine transport (Li et al., 2015). Cadaverine, putrescine, spermine, spermidine and acrolein have been tested as inhibitors of OCTs in immortalized human proximal tubule epithelial cell line (ciPTEC) on the uptake of the fluorescent OCT substrate, 4-(4-(Dimethylamino)styryl)-N methylpyridinium-Iodide (ASP^+^) (Schophuizen et al., 2013). Putrescine was the least potent inhibitor even though reported as an OCT substrate (Cunha et al., 2022). From homology models, α-helices involved in the recognition and in transport have been proposed. It would be interesting to reevaluate these findings with respect to the recently solved tridimensional structure of OCTs (Zeng et al., 2023).

#### 2.6.2 SLC22A4 (OCTN1)

SLC22A4 (OCTN1) is well known for its involvement in chronic inflammatory disorders. The OCTN1 variant L503F is associated with an increased susceptibility to Crohn’s disease. The first hypothesis concerning the ability of OCTN1 to transport polyamines derives from the observation that the OCTN1 variant L503F might increase the uptake of potential toxins, such as putrescine, derived from bacteria (Peltekova et al., 2004). Even though the physiological role of OCTN1 is not fully assessed, its association with inflammation is well recognized (Pochini et al., 2024). Among the most acknowledged substrates of OCTN1, there are acetylcholine and ergothioneine. The first is the player of the non-neuronal cholinergic system, besides its neurotransmitter role; the second is an exogenous antioxidant. Interestingly, the knockout OCTN1 mice apparently lack a phenotype. However, some differences among the wild type and knockout mice have been described after stress induction indicating that OCTN1 might play a role in recovery from stress (Pochini et al., 2022). The association of polyamines to prolonged life correlates well with the OCTN1 proposed role (Brito et al., 2022; Kurihara, 2022). OCTN1 may be involved in the transport of products of the gut microbiome. In this frame, it was not trivial to hypothesize that OCTN1 is involved in polyamine transport (Peltekova et al., 2004; Schophuizen et al., 2013). Indeed, the ability of OCTN1 to transport polyamine was recently demonstrated. Spermine present in inflamed intestinal tissue extracts was transported in HEK293 cells over-expressing OCTN1 (Masuo et al., 2018). Assays in proteoliposomes confirmed the ability of OCTN1 to interact with polyamines indicating a mixed inhibition by spermidine or spermine of the acetylcholine transport (Ki 35 ± 7.1 or 18 ± 2.4 μM) (Pochini et al., 2012). OCTN1 might also be involved in polyamine release (Pochini et al., 2015; Uehara et al., 2024). Accordingly, the link between polyamines and inflammation is also associated with OCTN1 function (Lian et al., 2022; Shenet al., 2023).

#### 2.6.3 SLC22A6

SLC22A6 (OAT1) mediates renal excretion of organic anionic and a few cationic drugs. Despite their cationic nature, spermidine and spermine were found to bind mouse OAT1 and are considered putative novel endogenous substrates. Starting with an integrated “omics”-driven network approach, the interaction of the polyamines with OATs has been investigated using adult kidney slices. Spermidine, spermine and arginine inhibited uptake of the OAT substrate, 6-carboxyfluorscein, 6CF (Ahn et al., 2011). Similar data were obtained from the *Xenopus* oocyte and CHO cell culture assay. Computational analysis confirmed the transcriptomic data. The collected data highlight a role for OAT1 in metabolism of polyamines (Liu et al., 2016).

#### 2.6.4 SLC22A16 (CT2)

SLC22A16 (CT2) is a carnitine transporter with a higher affinity for polyamine. The polyamine analogue, Bleomycin-A5 (BLM-A5), used in combination with other antineoplastic drugs to treat cancer (Aouida et al., 2010), is transported by CT2. According to CT2 sharp physiological localization in the epididymis and its over expression in specific cancers (NT2/D1 testicular cancer cells), some other cancers show resistance to BLM-A5. The availability of the reconstituted recombinant protein (Galluccio et al., 2022) will allow to assess the ability of CT2 to mediate polyamine transport.

#### 2.6.5 SLC22A18

In primary human melanocytes the mRNA expression level of SLC22A18 was decreased under spermidine treatment, as previously reported for SLC7A1 (Brito et al., 2022).

### 2.7 SLC44

The existence of a retina-to-blood transport system for spermine across the BRB was suggested (Kubo et al., 2014). Expression of SLC12A8 (CCC9) at this level has been found but its involvement in polyamine transport is questioned. This data together with the lack of inhibition by OCTs and CATs substrates allowed to exclude the involvement of CCC9, OCTs and SLC7 members in polyamine transport in this district. SLC44A1 (CTL1) has been suggested to be involved in putrescine excretion from the retina to the blood across the inner and outer BRB (Tega et al., 2023).

### 2.8 SLC47

The SLC47A1, multidrug and toxic compound extrusion (MATE) transporter 1, an H^+^/cation antiporter, is critical in the efflux of various organic cations from the brush-border and canalicular membrane of the kidney and liver, respectively. Considering that OCT and MATE transporters share overlapping substrate-specificity for several cationic compounds, polyamine transport was investigated. In HEK293 cells agmatine accumulation, in contrast to putrescine, was significantly enhanced by hMATE1 (Winter et al., 2011; Higashi et al., 2014).

### 2.9 Other putative polyamine SLCs

Arginine, the cardioprotective biomarker L-homoarginine and the uremic toxin asymmetric dimethylarginine, ADMA are substrates of the human renal transporter, SLC21/SLCO4C1 OATP4C1. L-homoarginine can also be exported by OATP4C1 out of cells (Taghikhani et al., 2019). Moreover, the involvement of SLC43A1 (along with SLC7A1) in Cav-1-dependent polyamine uptake has been hypothesized. Cav-1 is involved in the regulation of polyamine uptake in vascular smooth muscle cells (VSMC). Cav-1 KO VSMCs express higher levels of SLC43A1 mRNAs compared with WT cells (Grossi et al., 2014).

## 3 Conclusion

The issue of polyamine transport is becoming to be dealt with. Indeed, understanding the mechanisms of transport of these vital cations is critical in light of novel therapeutic approaches to human pathologies. Polyamine blockade therapy (PBT) is emerging as a novel adjuvant therapy of both chemo- and immune-therapies for a variety of cancers (Casero et al., 2018; Chin et al., 2022; Lian et al., 2022). Interestingly, the use of polyamine transport inhibitors (PTI) in combination with the ODC inhibitor α-difluoromethylornithine (DMFO), which is a largely used anticancer drug (Riviere-Cazaux et al., 2023), is promising (Lian et al., 2022). However, how these PTIs inhibit the transport systems and which specific transporter they inhibit is so far unknown (Dobrovolskaite et al., 2022). AMXT and other agents are effective in inhibiting polyamine uptake. It has been speculated that intracellular spermidine production might promote proliferation, whereas administration might activate the immune response overriding the tumor-promoting function (Zimmermann et al., 2023). Thus, from a different point of view, polyamine transport can be considered as a mean of delivering polyamine-conjugates or polyamine drug-like molecules to cells (Liu et al., 2019; Basagni et al., 2023) to exploit the self-regulation of polyamine homeostasis as a promising strategy for therapeutic benefit in neoplastic conditions (Ma et al., 2020; Holbert et al., 2022; Lodeserto et al., 2022; Zhang et al., 2022). For all these possible therapeutic approaches, many open questions concerning the transport of polyamines require urgent answers. Indeed, only a deep knowledge of the transport mechanisms and the structure/function relationships of the SLCs involved in polyamine transport will allow the design of novel molecules for the treatment of the alteration of polyamine homeostasis. The recently solved structures of some SLC22 transporters hypothesized to be involved in polyamine transport could fuel efforts in this direction.
